# Bioremediation of Synthetic Dyes by White-Rot Fungi: Enzymatic Mechanisms, Biosorption, and Environmental Applications

**DOI:** 10.3390/molecules31071085

**Published:** 2026-03-26

**Authors:** Anna Carolina Bruno Ferreira, Ygor Velloso Tavares, Nina Rezende Fontana, Thiago Machado Pasin, Carlos Adam Conte-Junior, Alex Graça Contato

**Affiliations:** 1 Analytical and Molecular Laboratorial Center (CLAn), Institute of Chemistry (IQ), Federal University of Rio de Janeiro (UFRJ), Cidade Universitária, Rio de Janeiro 21941-909, RJ, Brazil; annacarolinabrunofe@gmail.com (A.C.B.F.); vellosoygor@gmail.com (Y.V.T.); ninarfontana@eq.ufrj.br (N.R.F.); conte@iq.ufrj.br (C.A.C.-J.); 2 Center for Food Analysis (NAL), Technological Development Support Laboratory (LADETEC), Federal University of Rio de Janeiro (UFRJ), Cidade Universitária, Rio de Janeiro 21941-598, RJ, Brazil; 3 Laboratory of Advanced Analysis in Biochemistry and Molecular Biology (LAABBM), Department of Biochemistry, Federal University of Rio de Janeiro (UFRJ), Cidade Universitária, Rio de Janeiro 21941-909, RJ, Brazil; 4 Federal Institute of Education, Science and Technology of Rio de Janeiro (IFRJ), Realengo, Rio de Janeiro 21710-240, RJ, Brazil; 5 Graduate Program in Biochemistry (PPGBq), Institute of Chemistry (IQ), Federal University of Rio de Janeiro (UFRJ), Cidade Universitária, Rio de Janeiro 21941-909, RJ, Brazil; 6 Department of Oral Biology, College of Dentistry, University of Florida, Gainesville, FL 32611, USA; TMachadoPasin@dental.ufl.edu; 7 Graduate Program in Food Science (PPGCAL), Institute of Chemistry (IQ), Federal University of Rio de Janeiro (UFRJ), Cidade Universitária, Rio de Janeiro 21941-909, RJ, Brazil; 8 Graduate Program in Veterinary Hygiene (PPGHV), Faculty of Veterinary Medicine, Fluminense Federal University (UFF), Niterói 24220-000, RJ, Brazil; 9 Graduate Program in Chemistry (PGQu), Institute of Chemistry (IQ), Federal University of Rio de Janeiro (UFRJ), Cidade Universitária, Rio de Janeiro 21941-909, RJ, Brazil; 10 Department of Agricultural, Livestock and Environmental Biotechnology, Faculty of Agricultural and Veterinary Sciences (FCAV), Sao Paulo State University (UNESP), Jaboticabal 14884-900, SP, Brazil

**Keywords:** bioremediation, ligninolytic enzymes, synthetic dyes, textile industry, white-rot fungi

## Abstract

The widespread utilization of synthetic dyes within the textile industry, driven by their chemical recalcitrance and diverse chromatic spectra, constitutes a significant global environmental challenge. Improper discharge of these highly stable effluents into natural water bodies leads to severe ecological imbalances, affecting aquatic life and soil integrity while posing indirect risks to human health due to their mutagenic potential. Conventional physicochemical treatment methods are often hindered by prohibitive operational costs and the frequent generation of hazardous secondary pollutants. Consequently, there is an urgent demand for sustainable biotechnological alternatives to mitigate these industrial impacts. Bioremediation, specifically using white-rot fungi, represents a robust and eco-friendly strategy for the degradation of complex aromatic structures. Species such as *Trametes versicolor*, *Pleurotus ostreatus*, and *Phanerochaete chrysosporium* utilize a specialized extracellular enzymatic complex to mineralize toxic compounds effectively. Here we review the ligninolytic capacity of white-rot fungi and their specialized enzymatic systems for environmental sustainability. The primary points are: (i) the biochemical mechanisms of the ligninolytic system of laccases and peroxidases during dye degradation; (ii) the influence of operational parameters such as pH, temperature, and nutrient availability on fungal metabolic efficiency; (iii) the diverse environmental applications of these microorganisms in treating real textile effluents; (iv) the current biotechnological challenges, including maintaining enzymatic stability in non-sterile industrial environments; and (v) the future perspectives for scaling up fungal treatment systems from laboratory research to large-scale industrial implementation.

## 1. Introduction

Synthetic dyes are widely used in the textile industry because they offer a great variability of colors, low cost, and high durability. However, their use is associated with a serious environmental problem [1]. It is estimated that about 20% of the dyes applied in dyeing do not adhere to the fibers during the assembly and fixation stages. Consequently, these compounds end up being released into industrial effluents, mainly during the continuous washing process [2]

Azo dyes, characterized by the presence of one to three azo bonds (R1-N=N-R2) as a chromophore group, are the largest class of dyes in the world due to their color variability and resistance, and they are commonly derived from aniline and aromatic rings [3]. In turn, anthraquinone dyes are derived from anthraquinone (three aromatic rings with C=O groups at positions 9 and 10 as a chromophore), and they are light-resistant and stable; however, they are expensive and have low dyeing efficiency compared to azo dyes. Reactive dyes are formed by covalent bonds with the fibers, are highly soluble in water, and possess good fastness, being resistant to fading and washing. This type of dye generates a high concentration of salts and unreacted dyes in the effluents because they require an alkaline medium to activate the reaction [4,5].

The released effluents also contain agents in their composition that assist in the process, such as heavy metals, acids, and salts [6,7]. These substances are mostly xenobiotic and harmful to the organisms present in the environment. In aquatic ecosystems, these dyes color the water and prevent sunlight from penetrating, inhibiting the photosynthesis of phytoplankton and aquatic plants. Furthermore, the organic matter present in the composition of these effluents stimulates the excessive growth of algae, causing hypoxia and the death of organisms [8].

Heavy metals associated with synthetic dyes in the industrial process can also infiltrate the soil and reach groundwater, reducing the quality of underground water and soil fertility and permeability, remaining sedimented for a long time [9]. Thus, since dyes are foreign substances to biological systems and recalcitrant—that is, they cannot be easily metabolized—it is necessary to seek methods that remove them from the ecosystem and, preferably, have a low environmental impact to mitigate environmental pollution. Some techniques, such as adsorption and chemical degradation, are efficient for removing the coloration left by these effluents in water; however, the issues related to high costs and increased waste in the effluents make them less viable [8]. Coagulation/flocculation uses an excess of polyelectrolyte (such as Al_2_(SO_4_)_3_) to achieve high efficiency and is not efficient for treating effluents with water-soluble dyes such as reactive and acid dyes; therefore, techniques like bioremediation are preferable for treating pollutants [9].

Bioremediation is a biotechnological process that uses living organisms, such as microorganisms (bacteria and fungi), plants, or enzymes, to degrade, transform, or remove toxic pollutants from the environment, such as soils, groundwater, or sediments [10]. The organisms convert harmful substances into less toxic or harmless compounds to promote environmental restoration in a sustainable and economical manner [11]. This technique depends on factors such as the presence of microorganisms capable of degrading the compounds, temperature, pH, soil, and nutrients. Among the several microorganisms used in bioremediation processes, white-rot fungi (WRF) have garnered great interest due to their ability to degrade a wide variety of recalcitrant organic compounds, including synthetic dyes, as they are among the few microorganisms capable of breaking down highly recalcitrant and structurally complex organic polymers present in plant cell walls, making them efficient agents for the biodegradation, biotransformation, and mineralization of these dyes [12].

In this context, the present literature review aims to analyze and systematize the use of WRF in the bioremediation of synthetic industrial dyes, with emphasis on the mechanisms involved, the main ligninolytic enzymes, and the conditions that influence process efficiency. Several review studies have previously addressed the application of fungi in dye removal; however, many of them focus mainly on specific enzymatic systems, individual fungal species, or general bioremediation approaches. Despite the growing number of studies on this topic, the literature still presents gaps related to the comparative evaluation of different white-rot fungal species and the translation of results obtained at laboratory scale to industrial-scale applications. Furthermore, many studies address biochemical or environmental aspects in isolation, without a critical integration between enzymatic mechanisms and the technological potential of bioremediation. Therefore, a comprehensive analysis that integrates enzymatic mechanisms, fungal diversity, and operational conditions is still required to better understand the potential of WRF for the treatment of dye-contaminated effluents. In this sense, this review seeks to contribute to the advancement of the knowledge frontier by providing an integrated overview of recent advances in the use of WRF for the bioremediation of synthetic dyes, identifying current limitations, and highlighting perspectives for the development of more efficient, sustainable, and scalable processes for the treatment of industrial effluents containing synthetic dyes.

## 2. White-Rot Fungi as Bioremediation Agents

**Figure 1:**
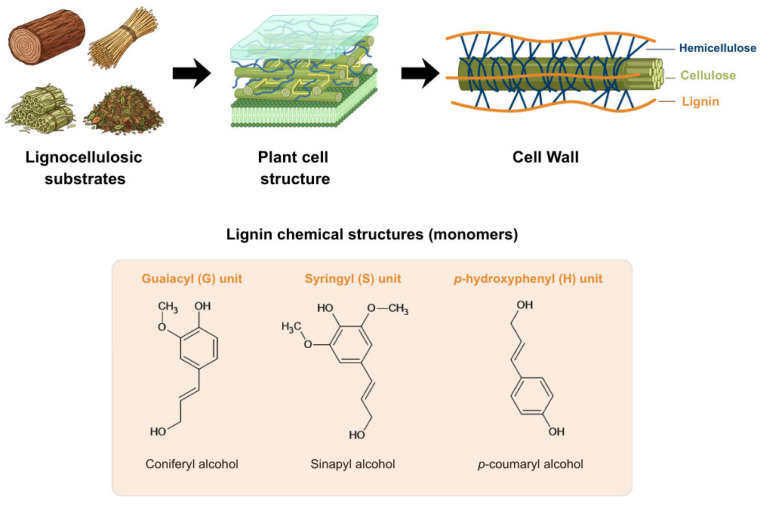
Schematic representation of lignocellulosic biomass and its structural organization at different levels. Natural lignocellulosic substrates (such as wood, straw, bagasse, and forest litter) are shown, followed by the hierarchical organization of the plant cell structure and the plant cell wall. The cell wall is composed mainly of cellulose microfibrils embedded in a matrix of hemicellulose and lignin. The figure also illustrates the main lignin monomers: guaiacyl (G), syringyl (S), and *p*-hydroxyphenyl (H) units, derived respectively from coniferyl, sinapyl, and *p*-coumaryl alcohols.

The use of fungi to degrade xenobiotic effluents is a promising alternative that does not generate an environmental impact. White-rot fungi are a group of microorganisms belonging to the phylum Basidiomycota, known for their ability to degrade the main components of the plant cell wall: cellulose, hemicellulose, and lignin. In this context, Figure 1 provides a visual overview of the structural complexity of lignocellulosic materials targeted by these fungi, illustrating how cellulose fibers are protected by hemicellulose and lignin within the plant cell wall, as well as the molecular diversity of lignin through its main monomeric units. Lignin is a complex, three-dimensional biopolymer formed by the polymerization of the monolignols coniferyl alcohol, *p*-coumaryl alcohol, and sinapyl alcohol, corresponding to the guaiacyl (G), *p*-hydroxyphenyl (H), and syringyl (S) subunits, respectively [13].

**Figure 2:**
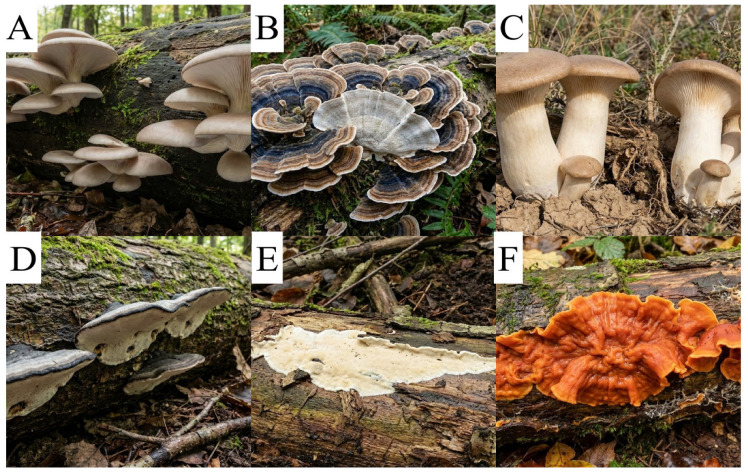
The main species of white-rot fungi. (**A**): *Pleurotus ostreatus* (Oyster mushroom), (**B**): *Trametes versicolor* (Turkey tail), (**C**): *Pleurotus eryngii* (King oyster mushroom), (**D**): *Bjerkandera adusta* (Smoky bracket), (**E**): *Phanerochaete chrysosporium* (White-rot crust fungus), and (**F**): *Phlebia radiata* (Wrinkled crust). Images generated using Nano Banana Pro 2 (Gemini) based on user-defined morphological parameters.

This characteristic provides them with great ecological relevance and high biotechnological potential in environmental bioremediation processes; such potential arises from the fact that lignin is a complex, highly recalcitrant heteropolymer responsible for providing structural rigidity to plants. Due to the inherent resistance of this polymer to degradation, the ligninolytic enzyme systems secreted by these fungi have become primary targets for research into the biodegradation of pesticides, phenolic compounds, and other xenobiotics, including the synthetic industrial dyes azo, anthraquinone, and triarylmethane dyes, also showing high efficiency in decolorization. The main species highlighted as bioremediation agents are *Bjerkandera adusta*, *Phanerochaete chrysosporium*, *Phlebia radiata*, *Pleurotus eryngii*, *Pleurotus ostreatus* and *Trametes versicolor* (Figure 2) [14,15].

*P. chrysosporium* is considered a model of WRF because it was one of the first to be used in biodegradation and is widely used in biotransformation techniques due to the ligninolytic capacity of its enzyme complex [16]. The ligninolytic enzymes produced by this fungus are lignin peroxidases (LiPs) and manganese peroxidases (MnPs). Their expression depends on factors such as the presence of nitrogen in the culture medium, pH, nutrient availability, and temperature [17]. Furthermore, the corticioid species *P. radiata* has been studied for the expression of genes and enzymes associated with lignocellulose decomposition [18]. Environmental and nutritional factors, such as copper availability, nitrogen levels, and wood supplementation, regulate the production of ligninolytic enzymes secreted by this fungus, including lignin peroxidase, manganese peroxidase, and laccase [19,20].

Also notable in enzyme production are the fungi within the genus *Pleurotus*, notably *P. ostreatus* and *P. eryngii*, which are particularly prominent due to their ease of cultivation and high yield of ligninolytic enzymes [21]. These fungi can produce laccase enzymes, belonging to the class of polyphenol oxidases, with the main function of oxidizing phenolic and non-phenolic compounds through the reduction of molecular oxygen to water. In addition, they are also responsible for the oxidation of Mn^2+^ to Mn^3+^, and their expression is also conditioned by certain factors, the main one being copper ions (Cu I, II, III, and IV) [22,23]. Furthermore, they are edible and can be cultivated on a wide range of agricultural residues, showing tolerance to variations in temperature and humidity. The ligninolytic system of both fungi consists of laccase, manganese peroxidase and versatile peroxidase [24,25], which have been applied in the bioremediation of dyes and phenolic compounds [26,27]. Reinforcing their biotechnological relevance, laccase production in *Pleurotus* species can be significantly enhanced by optimizing cultivation conditions, especially through the use of low-cost agro-industrial residues as substrates [28].

Other extensively researched basidiomycetes include *T. versicolor* and *B. adusta*, lignicolous fungi characterized by the formation of coriaceous basidiomata and high efficiency in the degradation of aromatic compounds and by the production of laccases and peroxidases [29,30]. In addition, these fungi also produce extracellular oxidases capable of generating H_2_O_2_ required for peroxidase activity, including aryl alcohol oxidase (AAO), as well as other enzymes, such as pyranose oxidase, glucose oxidase, and glyoxal oxidase. These oxidases play an important role in ligninolytic systems by supplying H_2_O_2_, which acts as the essential co-substrate for lignin peroxidase, manganese peroxidase, and versatile peroxidase during the oxidative degradation of complex aromatic compounds [31,32]. Another enzyme produced is dye-decolorizing peroxidase (DyP), a heme-containing peroxidase, involved in the decolorization of dyes and phenolic and non-phenolic substrates [33,34].

## 3. Oxidative Enzymes

Since these enzymes play an important role, it is necessary to understand their mechanisms of action on the substances and the factors that stimulate/inhibit their expression. Lignin peroxidase, manganese peroxidase, and dye-decolorizing peroxidase belong to the same class of enzymes, and all possess the heme prosthetic group (active center responsible for the redox reactions that sustain their catalytic activity) in their structure, thus playing a fundamental role in lignin degradation. The dye-decolorizing peroxidase (DyP) activity consists of the decolorization of dyes and phenolic and non-phenolic substrates. In addition, these fungi also produce aryl alcohol oxidase (AAO), which participates in the process by contributing to the production of H_2_O_2_ for the peroxidases to act on and to the oxidation of substrates, and laccases, which are multicopper oxidases that catalyze the one-electron oxidation of phenolic or aromatic substrates, coupling these reactions to the four-electron reduction of O_2_ to water [32,33,34,35].

Table 1 provides an overview of the main ligninolytic and oxidative enzymes produced by the white-rot fungi, their catalytic functions and the representative fungal species known to express these enzymatic systems. All of these enzymes are fundamental for lignin degradation, aromatic compound oxidation, and decolorization of dyes that characterize the importance of white-rot fungi in environmental, industrial, and biotechnological contexts.

### 3.1. Peroxidases

**Figure 3:**
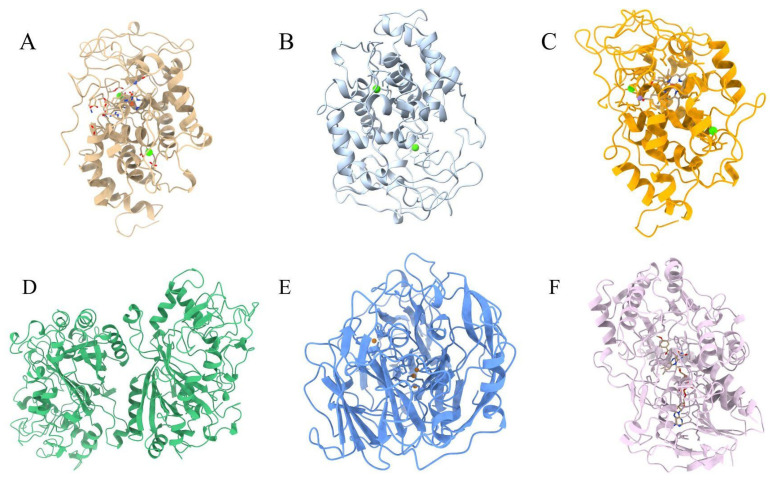
Three-dimensional homology models of enzymes analyzed in this review. Models were generated by homology modeling using the SWISS-MODEL server based on amino acid sequences retrieved from the UniProt database: (**A**) lignin peroxidase (UniProt accession P11542), (**B**) manganese peroxidase (UniProt accession Q02567), (**C**) versatile peroxidase (UniProt accession Q9UR19), (**D**) dye-decolorizing peroxidase (UniProt accession Q0VTU1), (**E**) laccase (UniProt accession Q12739), and (**F**) aryl-alcohol oxidase (AAO) (UniProt accession O94219). In panel (**A**), two calcium ions are shown in green with the heme prosthetic group. In panels (**B**,**C**), two calcium ions are represented in green; in panel (**C**), the manganese ion is shown in lilac with the heme prosthetic group. In panel (**E**), four copper ions forming the multicopper catalytic center of laccase are represented in orange. In panel (**F**), the flavin adenine dinucleotide (FAD) cofactor and a generic nitrogen-containing ligand (AAN) introduced during homology modeling are shown. Structures were visualized using UCSF ChimeraX 1.11.

Lignin peroxidase (EC 1.11.1.14) is one of the enzymes secreted by filamentous fungi; like all peroxidases, it uses hydrogen peroxide (H_2_O_2_) as an oxidizing agent to break the bonds of lignin and performs electron transfer during the oxidation of aromatic and non-aromatic phenolic compounds [51]). Among the substrates reduced by it, the most important is veratryl alcohol (VA, 3,4-dimethoxybenzyl alcohol), a secondary metabolite of *P. chrysosporium*, which is a mediator in the oxidation of other compounds. Regarding the structure, according to [52], it is composed of 343 to 344 amino acids, depending on the isoenzyme (LiP H1 to LiP H10), and contains two Ca^2+^ ions, in addition to the heme prosthetic group, which are highlighted in the structural representation shown in Figure 3A [44,52].

Manganese peroxidase (EC 1.11.1.13) is a heme peroxidase, like LiP, but differs in its high specificity for Mn^2+^ and its ability to oxidize this ion to Mn^3+^ in the presence of H_2_O_2_. The catalytic cycle begins with the native Fe^3+^ enzyme, which reacts with H_2_O_2_ to form a heme–peroxide intermediate. The cleavage of the peroxide bond leads to the formation of Compound I (Fe^4+^=O + porphyrin radical), a highly oxidizing intermediate. Compound I oxidizes Mn^2+^ to Mn^3+^, producing Compound II (Fe^4+^=O). A second molecule of Mn^2+^ donates an electron to Compound II, producing additional Mn^3+^ and regenerating the native state of Fe^3+^, thus completing the catalytic cycle. The produced Mn^3+^ is stabilized by organic acids and functions as a diffusible redox mediator capable of oxidizing phenolic compounds, lignin fragments, and other aromatic substrates. Structurally, MnP is a stable enzyme that contains five disulfide bridges and two essential Ca^2+^ ions to maintain the integrity of the active site [53]. These structural and functional features of MnP are illustrated in Figure 3B. The literature also reports that isoenzymes of *P. eryngii* and *B. adusta* exhibit a broader substrate specificity, being capable of oxidizing non-phenolic compounds and, therefore, functionally resembling LiPs [54]).

Versatile peroxidase (EC 1.11.1.16) is considered one of the most important enzymes involved in the degradation of lignin and other recalcitrant compounds. It was first described in the fungus *P. eryngii* and is regarded as a hybrid enzyme of LiP and MnP, as it combines catalytic properties that enable the oxidation of non-phenolic aromatic compounds with high redox potential, as well as the oxidation of Mn^2+^, dyes, and both phenolic and non-phenolic aromatic compounds [55,56,57]. These dual catalytic features are reflected in the structural model shown in Figure 3C, which highlights the presence of the heme prosthetic group, two essential calcium ions involved in structural stabilization, and the manganese binding site, illustrating the structural basis for the combined LiP and MnP activities of VP.

Dye-decolorizing peroxidase (EC 1.11.1.19) belongs to the heme peroxidase family and is found in bacteria and ligninolytic fungi. The presence of DyPs is common in white-rot fungi, such as *T. versicolor* and *P. ostreatus*, and these enzymes are widely reported in the degradation of lignin, the oxidation of phenolic and non-phenolic aromatic compounds, and the decolorization of several industrial dyes, mainly those derived from anthraquinone [39,58]. However, some isoforms, such as TvDyP1 studied by Amara et al. [58], also show activity on azo dyes, indicating a certain versatility. In the study, the authors analyzed the DyP and VP secreted by *T. versicolor* through heterologous expression in *Escherichia coli*. They evaluated the ability of these enzymes to decolorize different dyes and oxidize complex substrates under acidic conditions. Among the compounds tested were ABTS, RB5 (azo dye), and RB19 (anthraquinone dye). They concluded that TvDyP1 exhibits high stability at acidic pH and temperatures between 30 and 50 °C, maintaining between 20 and 80% of its activity, but the enzyme did not show good decolorization activity for all the dyes tested, although it did show significant decolorization for Acid Black 172 (AB), an azo dye, and also a 1:2 metal complex. Thus, despite its interesting biochemical characteristics and potential for treating synthetic dyes, the limited range of substrates indicates that further studies are still needed to better understand the restrictions of its decolorizing activity. Thus, Figure 3D illustrates the heme-dependent structure of DyP associated with the oxidation of aromatic compounds and dye decolorization.

### 3.2. Laccase

Laccases (EC 1.10.3.2) are multicopper oxidases present in ligninolytic fungi; they catalyze the oxidation of phenolic and aromatic compounds and reduce molecular oxygen to water without the need for hydrogen peroxide, unlike the others [59]. Regarding the factors influencing the production of these enzymes, bacterial laccases tend to perform better in more alkaline pH ranges, whereas fungal laccases exhibit higher activity in acidic media, typically between 3 and 6 [60]. Ramayanam [61] developed a sustainable strategy to optimize extracellular laccase production by *T. versicolor*, utilizing low-cost cultivation and supplementation approaches. The author reports that at a temperature of 30 °C and pH 5.5 and with the addition of 0.2 mM CuSO_4_, the fungal yield can be doubled. Furthermore, the degradation of azo bonds and a 99% reduction in the color of real textile dye bath effluent were observed, reaffirming its potential as an eco-friendly treatment for wastewater. Similarly, *Trametes* sp. M3 has been reported as a promising agent for textile effluent treatment due to its ability to decolorize and detoxify the synthetic dye Reactive Blue 268 [62].

In addition, studies such as Benavides et al. [63] investigate how the supplementation of metals (copper and manganese) in WRF cultures can influence enzymatic activity, specifically Lac and MnP. It was concluded that the addition of metals is an effective optimization strategy; however, precise concentrations must be applied, as excess may inhibit production. An increase of over 90% in the activity of these enzymes was observed when doses of 0.1–100 mM and 0.2–18.2 mM were added, respectively, compared to the controls. Moreover, it is not possible to define an optimal concentration of these metals, as it remains highly dependent on the substrate used.

In structural terms, laccase contains four copper ions distributed across three distinct catalytic centers (T1, T2, and T3) that act to promote electron transfer [64]. Copper T1 acts as the main substrate oxidation site and gives the enzyme its blue color, while the T2/T3 centers are involved in the reduction of molecular oxygen. As shown in Figure 3E, the four copper ions form a compact multicopper center that enables efficient electron transfer between the substrate and molecular oxygen. This enzyme can oxidize aromatic and non-aromatic compounds through a mechanism that involves radicals; these same radicals can undergo other reactions, such as polymerization [65].

An important strategy to expand the oxidative capacity of laccases is the use of laccase–mediator systems (LMSs). In these systems, low-molecular-weight redox mediators act as electron shuttles between the enzyme and substrates that cannot be directly oxidized due to steric or redox potential limitations. After being oxidized by laccase, the mediator forms reactive radical intermediates capable of attacking complex dye molecules, significantly increasing the range of degradable compounds [66]. Several synthetic mediators, such as 1-hydroxybenzotriazole (HBT), ABTS, and violuric acid, have been widely investigated, as well as natural mediators derived from lignin degradation [67,68,69]. Numerous studies have demonstrated that LMSs can substantially enhance the decolorization of recalcitrant dyes, including azo, anthraquinone, and indigoid dyes [70,71,72,73]. However, mediator toxicity, cost, and stability remain important challenges that must be considered when applying these systems in large-scale wastewater treatment [74].

### 3.3. Aryl Alcohol Oxidase

Aryl alcohol oxidase (EC 1.1.3.7) belongs to the glucose–methanol–choline (GMC) oxidoreductase family, a group of FAD-dependent enzymes widely distributed in ligninolytic fungi. It was first identified by Farmer et al. [75] in the culture medium of *Polystictus versicolor*, now known as *Trametes versicolor*. As lignin degradation involves the participation of hydrogen peroxide-dependent enzymes, it produces the hydrogen peroxide necessary to activate the peroxidases of ligninolytic fungi through the oxidation of aromatic and aliphatic alcohols [76]. Moreover, aromatic alcohols and aldehydes derived from lignin may participate in extracellular redox cycling reactions that contribute to sustained peroxide production during lignin degradation [77]. In Figure 3F, the homology model reveals the location of the FAD cofactor in the active site, which underlies the enzyme’s oxidative function and its contribution to hydrogen peroxide production in ligninolytic fungi [32].

While the enzymes described above have been extensively studied individually, their effectiveness in dye removal often depends on the chemical structure of the target compound and on the physicochemical conditions of the wastewater. Therefore, from an application-oriented perspective, it is also useful to consider how different classes of dyes typically respond to fungal enzymatic systems. In general, azo dyes are frequently degraded through reductive cleavage of the azo bond followed by oxidative transformations [78], whereas anthraquinone and triarylmethane dyes often require strong oxidative enzymes such as laccases or peroxidases [79,80]. However, the efficiency of these processes can be significantly influenced by environmental factors, including pH, salinity, chloride concentration, the presence of metal ions, mediator availability for laccases, and the control of H_2_O_2_ levels in peroxidase-based systems. For practical purposes, some general trends reported in the literature are summarized in Table 2.

## 4. Mechanisms of Dye Biodegradation

**Figure 4:**
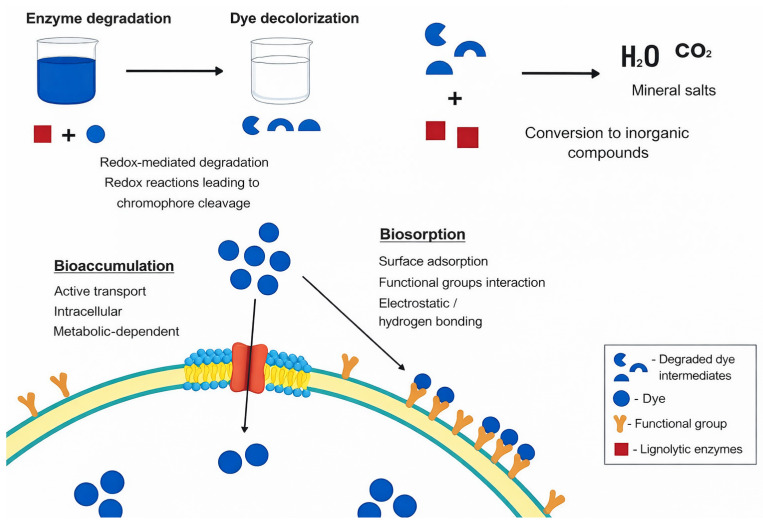
Schematic representation of the main mechanisms involved in synthetic dye removal by fungal systems, including enzymatic degradation, mineralization, biosorption on the cell surface, and intracellular bioaccumulation.

The biodegradation of synthetic dyes through ligninolytic enzymes is of great interest for effluent treatment because these enzymes can oxidize complex dye molecules and cleave chromophoric structures [90]. However, depending on the degradation pathway, intermediate compounds such as aromatic amines may be formed, which can exhibit higher toxicity than the parent dye before further transformation or mineralization [78]. The mechanisms involved in this chemical transformation process include both physical and biological processes that can occur complementarily, such as bioaccumulation, biosorption, enzymatic degradation, and mineralization, as schematically summarized in Figure 4.

As summarized in Table 3, bioaccumulation and biosorption are associated with dye removal without chemical modification, whereas enzymatic degradation and mineralization involve the transformation and complete breakdown of dye molecules.

Bioaccumulation is an active process that occurs in living fungal biomass, where compounds are absorbed into the interior of the cell. This process requires the presence of nutrients, is controlled by cellular metabolism and is partially reversible. As conceptually illustrated in Figure 4, bioaccumulation is part of the overall uptake mechanisms, where dye molecules are internalized by the fungal biomass prior to enzymatic transformation or degradation [91,96].

Although there is strong evidence of intracellular bioaccumulation of dyes in filamentous fungi such as *Aspergillus niger* and *Penicillium funiculosum*, the current literature does not provide clear results showing intracellular bioaccumulation of synthetic dyes in WRF [92,97]. However, Barnhart-Dailey et al. [98] reported that the lignolytic organism *P. chrysosporium* and bacterium *Enterobacter lignolyticus* actively internalized and accumulated aromatic breakdown products (mono- and di-aryl compounds) through both lignolytic and non-lignolytic mechanisms via energy-dependent transport.

On the other hand, biosorption is a passive process that occurs in dead or inactivated biomass, where pollutants are simply adsorbed onto the cell surface. Unlike bioaccumulation, biosorption does not require nutrients, is not controlled by metabolism, is fully reversible, and poses no risk of toxic effects [91]. As depicted in Figure 4, this mechanism involves surface-level interactions between dye molecules and functional groups present in the fungal cell wall. An example of this process is the biosorption of triarylmethane dyes by *T. versicolor* (CB8) and *P. ostreatus* (BWPH), as reported by Upadhyay et al. [15]. In this mechanism, FT-IR analysis suggested that the binding of dyes to biomass occurred through hydrogen bonds and electrostatic interactions with the amine and hydroxyl functional groups present in the fungal cell wall. Moreover, the results demonstrated that the sponge-immobilized, live fungal biomass removed up to 90.3% of the dye Brilliant Green and 43.9% of Crystal Violet within 6 h, a performance five times better than self-immobilized biomass. This highlights the potential of WRF biomass and the sustainability of the method, since the immobilized biosorbents were reused without the need for additional treatment [15].

In addition, enzymatic degradation is a promising and widely applied approach due to its sustainability, high specificity, and versatility, as it can be employed either as a standalone process or in combination with other microorganisms and treatment methods. As schematically reflected in Figure 4, this mechanism is driven by ligninolytic enzymes responsible for the oxidative cleavage of dye structures. For example, the results obtained by Mostafa et al. [93] in an experiment evaluating the ability of the fungus *Cylindrocephalum aurelium* RY06 to decolorize dye demonstrated that the biodegradation of the dye Mordant Orange-1 (MO-1) by the fungus was highly efficient, promoting both the decolorization of the dye and its transformation into simpler, less toxic compounds in the environment. During the process, the enzymes Lac, LiP, MnP, 1,2-dioxygenase, and 2,3-dioxygenase were identified, with Lac showing the highest oxidative activity and playing a central role in the degradation. An analysis by GC-MS (gas chromatography coupled with mass spectrometry) revealed that MO-1 was biotransformed through two main metabolic pathways, resulting in maleic acid and isophthalic acid as final products. Additionally, intermediates such as 4-nitroaniline and 5-amino-2-hydroxybenzoic acid were detected, indicating that the dye undergoes an asymmetric cleavage. It has also been reported that this cleavage can occur in a symmetric or asymmetric manner, depending on the structure of the substrate and the enzymes involved in the enzymatic degradation process.

Mineralization is a process that aims to convert textile dyes into inorganic substances and less toxic compounds, such as CO_2_, H_2_O, and mineral salts, as depicted in Figure 4 [95]. Despite its relevance, achieving complete mineralization remains a challenge, as evidenced by experimental studies. Wanderley et al. [99] investigated the biodegradation of the azo dye Congo Red by *P. chrysosporium* in sequential batch bioreactors and observed significant dye decolorization due to the rupture of the azo bond (-N=N-). However, the mineralization of the compounds was limited and influenced by the dye/glucose ratio, and the degree of mineralization observed, particularly in reactor R2, was low and did not correspond to the complete conversion of organic compounds into final inorganic products. The use of bioreactors, including Sequencing Batch Reactors (SBRs), allows precise control of operational parameters such as pH, aeration, agitation, and nutrient availability, which are essential for maintaining microbial metabolic activity, maximizing compound oxidation, and enhancing process efficiency, enabling potential industrial application [94,99].

The results obtained demonstrate that although the system was efficient in color removal, dye mineralization was limited. Therefore, further studies are needed to optimize operating conditions and the use of co-substrates to avoid partial degradation of molecules and promote the mineralization of intermediates.

## 5. Biosorption by Fungal Biomass

**Figure 5:**
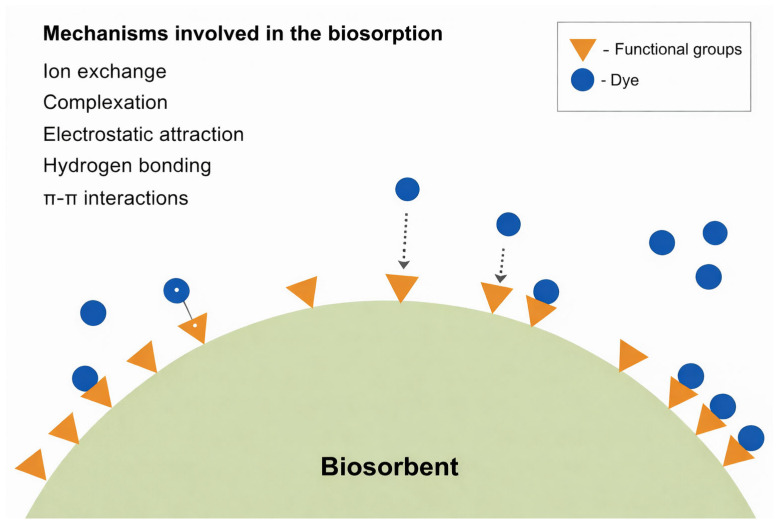
Schematic representation of the mechanisms involved in dye biosorption by the biosorbent, including ion exchange, complexation, electrostatic attraction, hydrogen bonding, and π-π interactions between surface functional groups and dye molecules.

One of the mechanisms involved in contaminant removal is biosorption, an ecological and low-cost alternative related to the removal of harmful compounds (such as metal ions and dyes) in effluents without generating metabolites [100]. Biosorption is a process that uses fungal biomass, either live or inactivated, to remove synthetic dyes through physical and chemical mechanisms. This phenomenon occurs due to the natural ability of fungal cell walls to bind contaminant ions and molecules, acting as true natural “biosorbents” (Figure 5). This behavior has been experimentally demonstrated in WRF, as previously reported by Upadhyay et al. [15]. In fungal biomass, dye adsorption is primarily associated with specific structural components of the cell wall. The fungal cell wall is a complex matrix mainly composed of chitin, chitosan, β-glucans, proteins, and lipids, which provide a variety of functional groups, such as amino, hydroxyl, carboxyl, and phosphate groups. These functional groups act as binding sites for dye molecules through mechanisms including electrostatic interactions, hydrogen bonding, van der Waals forces, and π–π interactions. Chitin and chitosan, in particular, play a key role due to the presence of amino groups that can interact strongly with anionic dyes, while glucans and associated proteins contribute to the overall adsorption capacity and structural stability of the biomass. Therefore, the composition and physicochemical properties of the fungal cell wall are critical factors influencing biosorption efficiency. Further, the authors have highlighted the immobilization of fungal biomass as a strategy to enhance biosorption performance, as immobilized systems tend to provide greater structural stability, facilitate biomass recovery and allow biosorbent reuse. For example, the immobilization of *T. versicolor* and *P. ostreatus* resulted in improved removal efficiency and reusability in the biosorption of triarylmethane dyes, reinforcing the potential of this approach for more robust and scalable wastewater treatment applications. Biosorption can be performed using either live biomass, in which contaminant removal may involve metabolic processes such as bioaccumulation, or dead (inactivated) biomass, where the phenomenon is entirely passive and depends solely on the chemical properties of the cell wall [91].

The use of dead fungal biomass offers advantages such as eliminating the need for nutrients, avoiding the generation of active biological waste, and providing greater resistance to environments with high contaminant concentrations [101]. The fundamental difference between biosorption and bioremediation lies in the type of process involved and the metabolic role of the organism. In biosorption, the process is essentially physicochemical and passive, occurring mainly on the cell surface and without active metabolic participation of the fungus. Al-Rajhi et al. [102] reported that non-viable *P. chrysosporium* biomass achieves removal efficiencies of up to 90% of the dyes Reactive Red and Reactive Blue from water. Also, temperature was shown to positively influence dye adsorption, suggesting an endothermic process in which higher temperatures enhance molecular diffusion and improve dye biosorbent interactions.

On the other hand, bioremediation involves metabolically active living organisms capable of transforming, degrading, or mineralizing toxic compounds into less harmful substances through enzymatic reactions [11]. In this case, there is direct involvement of microbial metabolism, which differentiates bioremediation from the simple adsorption of pollutants [100]. For example, while the biosorption of a dye occurs through its attachment to the fungal cell wall, bioremediation may involve the enzymatic degradation of this dye, breaking its chemical bonds and eliminating its toxicity [103]. Thus, it can be said that biosorption is a complementary or preliminary method of bioremediation, useful mainly for the rapid and efficient removal of contaminants from water, while bioremediation is a biodegradative process aimed at the complete transformation of the pollutant into non-toxic products [62,104].

Despite its advantages, biosorption also presents important limitations that must be considered when evaluating its effectiveness for dye removal. One of the main concerns is the potential desorption of previously adsorbed dyes, particularly under changing environmental conditions such as pH, ionic strength, or temperature fluctuations [105]. This reversibility can lead to the secondary release of pollutants into the treated effluent, limiting the long-term stability of the process.

Another critical aspect is the management of dye-loaded biomass generated after biosorption. Once saturated with contaminants, fungal biomass may require regeneration or safe disposal to avoid environmental risks. Although several regeneration strategies have been proposed, including chemical desorption or solvent washing, the efficiency of these approaches varies depending on the dye structure and the physicochemical characteristics of the biosorbent [106].

Furthermore, when reporting dye removal performance, it is essential to distinguish whether the observed removal efficiency is primarily due to adsorption (biosorption) or to enzymatic transformation and biodegradation. Since biosorption does not chemically alter dye molecules, removal percentages based solely on decolorization may overestimate the actual detoxification of the effluent [107].

For a more reliable comparison between studies, future research should also report additional parameters, such as biosorbent regeneration capacity, multi-cycle adsorption performance, the influence of salinity and competing solutes typically present in textile wastewater, and the potential for dye desorption under varying environmental conditions. Including these factors is essential for accurately assessing the practical applicability of biosorption processes in real wastewater treatment systems.

## 6. Environmental Applications and Case Studies

First, before detailing specific cases, it is worth highlighting that several studies have explored biotechnological strategies for wastewater treatment, revealing different approaches and results depending on the organism and the conditions used.

An early study conducted by Amaral et al. [108] investigated the decolorization of a mixture of textile dyes (Procion Orange MX-2R, Remazol Red 3B, and Remazol Black GF) using the fungus *T. versicolor*. In experiments with synthetic effluent, decolorization was more efficient in the presence of glucose, reaching up to 97% for concentrations of 50 and 100 mg L^−1^. Without glucose, Lac production was higher, but decolorization was lower. pH also affected the process, with values around pH 6 reducing decolorization by 50%, possibly due to the involvement of other ligninolytic enzymes or the need for metabolic mediators. For real effluent from the SENAI/CETIQT dyeing sector, decolorization was 40% at a sevenfold dilution (300 mg L^−1^) and increased to 92% at a 42-fold dilution (50 mg L^−1^). The lower initial efficiency was attributed to recalcitrant compounds, while pH and glucose consumption were similar, highlighting the importance of dilution to enhance the decolorization process. In addition, the efficiency reported in this study was evaluated based on color removal, which does not necessarily indicate complete degradation of dye molecules.

While earlier work by Amaral et al. [108] laid foundational insights into fungal dye decolorization, more recent research by Thampraphaphon et al. [109] expanded this understanding by investigating the efficiency of 16 isolates of WRF belonging to seven genera *(Amauroderma*, *Coriolopsis*, *Dentipellis*, *Ganoderma*, *Microporus*, *Pseudolagarobasidium*, *and Trametes*) in the decolorization of textile dyes from wastewater by MnP, using real textile wastewater with high salinity and containing heavy metals. The authors reported that *Trametes hirsuta* PW17-41 was the most efficient isolate, achieving more than 98% decolorization under optimized conditions. UV–visible and FTIR spectral analyses indicated that dye structural modification occurred in addition to color removal, suggesting enzymatic transformation of dye molecules mediated by the combined action of MnP and Lac enzymes. Notably, MnP played a central role in dye decolorization and transformation, differing from many previous studies that mainly reported Lac as the key enzyme in *Trametes*-based systems. These findings highlight the potential of *T. hirsuta* PW17-41 as an eco-friendly alternative for the treatment of complex textile wastewater, although further studies at bioreactor scale are required. Another study on *T. hirsuta*, conducted by Ortolan et al. [42], reported effective decolorization and increased Lac production by this fungus. The authors used orange waste as a substrate and achieved a significant increase in enzyme production, as well as effective decolorization of the dye Bromocresol Green, reaching nearly 97% decolorization within 24 h. The removal efficiency reported in this study was evaluated based on color reduction [42].

Therefore, the choice between using a single fungal species or a combination of species must be carefully considered to broaden the enzymatic repertoire and optimize degradation and decolorization processes. In the study conducted by Singh et al. [110], which evaluated the differences between monoculture and co-culture of ligninolytic fungi and their influence on enzyme production capacity, the fungi *P. ostreatus*, *T. versicolor*, *P. chrysosporium*, and *Daedaelia flavida* were investigated. Prior to co-cultivation, compatibility tests were performed, revealing antagonistic behavior among all fungal pairs, except for *T. versicolor* and *P. chrysosporium*. This compatible pair was able to grow on the same plate and rapidly decolorize the dye Reactive Blue MR (50 mg L^−1^). Such competitive and cooperative interactions among fungi can be strategically exploited to enhance ligninolytic enzyme production and dye decolorization performance, highlighting the importance of compatibility screening before establishing fungal consortia, since antagonistic interactions may limit treatment efficiency [110].

Moreover, the integration of white-rot fungi into physicochemical processes is also studied due to the limitations of bioremediation, such as sensitivity to pH; the need for substrates; temperature; and the presence of other recalcitrant compounds. This integration with methods such as flocculation, coagulation, adsorption, and advanced oxidative processes (AOPs) can increase the dye decolorization capacity, with some of the described advantages being increased efficiency in color and COD (Chemical Oxygen Demand) removal, reduced toxicity of the final effluent, and lower consumption of reagents and energy [111,112,113].

One of the well-known chemical methods is Fenton chemistry, which consists of oxidative reactions between iron ions and hydrogen peroxide that result in the formation of hydroxyl radicals (•OH). These radicals are highly reactive and capable of degrading complex organic compounds [114]. The synergy between lignin degradation and Fenton chemistry lies in the generation of hydroxyl radicals that promote lignin degradation by breaking its complex structure into simpler forms. This interaction is supported by the iron redox cycle (Fe^3+^/Fe^2+^), in which WRF promotes the regeneration of Fe^2+^ from Fe^3+^, thereby maintaining the Fenton reaction active. As a result, positive feedback occurs between chemical and biological processes, making lignin degradation more efficient than that achieved by each mechanism alone [115].

When van der Made et al. [115] investigated the synergistic lignin degradation between *P. chrysosporium* and Fenton chemistry, the results showed that the fungus alone degraded approximately 58.8% of lignin in 10 days, while Fenton chemistry alone degraded 92.3% only at high reagent concentrations. The combination of both methods resulted in approximately 80.4% lignin degradation even with low concentrations of Fenton reagents. Overall, these findings reinforce the conclusion that combining bioremediation strategies with physicochemical methods represents a promising approach for enhancing the efficiency of lignin degradation.

While biological processes offer selectivity and environmental compatibility, physicochemical methods provide rapid degradation of recalcitrant compounds. The combined application of bioremediation and physicochemical techniques emerges as a sustainable and efficient strategy for the treatment and valorization of lignocellulosic materials.

## 7. Challenges and Perspectives

Although bioremediation using fungi and their enzymes is a promising approach for the removal of recalcitrant and toxic compounds from the environment, several limitations still hinder its large-scale application. These include difficulties related to enzyme isolation, short enzyme lifespan, and challenges associated with process scale-up. Consequently, results obtained under controlled laboratory conditions are often difficult to reproduce when applied to real effluents. Therefore, it is essential to critically evaluate the challenges that restrict industrial implementation, as well as the factors that compromise the stability and efficiency of fungal systems, to overcome these obstacles.

1. Environmental conditions:

The performance of ligninolytic enzymes produced by white-rot fungi is highly dependent on environmental factors such as pH, temperature, nutrient availability, and humidity. These enzymes require specific conditions to achieve optimal catalytic activity, and deviations can significantly reduce their efficiency [116].

2. Effluent composition and dye concentration:

High dye concentrations can negatively affect the decolorization process. Amaral et al. [108] demonstrated that dilution of real textile effluent was necessary to achieve effective decolorization, highlighting the inhibitory nature of complex effluent matrices. The presence of metals used in the dyeing process of fabrics and other components present in the effluents is also a factor that hinders bioremediation.

3. Physiological variability of fungi:

White-rot fungi exhibit significant differences in growth rate, cultivation time, enzymatic complexes produced, substrate requirements, and tolerance to environmental stress. This variability complicates process standardization and scalability.

4. Use of synthetic *versus* real effluents:

Most studies evaluating fungal treatment of dye-containing wastewater are performed using synthetic dye solutions, which do not fully reflect the physicochemical complexity of real textile effluents. Consequently, treatment efficiencies observed under laboratory conditions may differ when applied to industrial wastewater. To illustrate this aspect, Table 4 compares selected studies employing fungal biomass or ligninolytic enzymes for dye removal in either synthetic or real effluents. Although these studies demonstrate the potential of fungal-based systems for dye degradation, the predominance of experiments conducted with synthetic matrices highlights an important research gap. Future investigations should therefore prioritize the use of real textile wastewater or more complex simulated effluents to better assess the robustness and practical applicability of these treatment strategies.

5. Strategies to overcome current limitations:

Recent advances in biotechnology and bioprocess engineering have provided promising strategies to overcome several limitations associated with fungal bioremediation systems. One of the most widely explored approaches is enzyme immobilization, which can significantly enhance enzyme stability, reusability, and resistance to adverse environmental conditions. Immobilized ligninolytic enzymes, such as laccases and peroxidases, have demonstrated improved catalytic performance and operational stability when applied to dye degradation processes [42,118,119].

Another important strategy involves the development of improved bioreactor configurations designed to optimize fungal growth and enzymatic activity. Reactor systems such as packed-bed reactors, fluidized-bed reactors, and membrane bioreactors have been investigated to enhance mass transfer, maintain favorable environmental conditions, and increase the efficiency of pollutant removal in continuous treatment processes [120].

In addition, advances in molecular biology and fungal biotechnology have opened new opportunities for improving the degradation capacity of fungal systems. Genetic engineering and heterologous expression of ligninolytic enzymes have been explored to increase enzyme production and catalytic efficiency [104]. Similarly, metabolic engineering approaches may enable the development of fungal strains with enhanced tolerance to toxic compounds and improved degradation pathways [121,122].

Together, these strategies highlight the potential for integrating biotechnological innovations with traditional fungal treatment systems, which may significantly improve feasibility and scalability.

## 8. Conclusions

Synthetic dyes are widely used worldwide due to their advantages, such as variability and durability. However, when improperly disposed of or inadequately treated, these compounds are released into ecosystems, causing significant harm to living organisms. This scenario highlights the urgent need for effective, low-cost treatment methods that minimize environmental impacts while promoting the removal of xenobiotic compounds and water decolorization. In this context, bioremediation has emerged as a promising strategy, particularly through the use of white-rot fungi. The application of these organisms, either alone or in combination with physicochemical processes, represents an environmentally sustainable alternative for the treatment of textile effluents, characterized by low operational cost and high bioavailability. Evidence indicates that the oxidative enzymes produced by white-rot fungi, particularly lignin peroxidase, manganese peroxidase, versatile peroxidase, dye-decolorizing peroxidase, and laccase, exhibit remarkable efficiency in the degradation of dyes, including azo, anthraquinone and reactive dyes, highlighting the central role of the ligninolytic enzymatic system in the oxidative breakdown of structurally complex chromophoric groups.

Despite limitations related to enzyme induction and secretion control, the fungal enzymatic system demonstrates a strong ability to degrade structurally complex pollutants, contributing to the recovery of environments impacted by textile industry discharges. Among the technological strategies discussed, the use of immobilized enzymes, fungal consortia, and hybrid treatment systems combining biological and physicochemical methods appears particularly promising for improving treatment performance. Nevertheless, additional studies under real-scale conditions are essential to validate the industrial applicability of these biotechnological strategies. Greater attention should also be directed toward the development of fungal consortia, particularly those involving compatible WRF species capable of establishing synergistic rather than competitive interactions. Such relationships may stimulate enzymatic secretion and improve pollutant degradation performance. Moreover, the use of low-cost substrates and agro-industrial extracts as enzymatic inducers represents a promising pathway to increase economic feasibility while maintaining high treatment efficiency. Advancing research in these areas, together with improved understanding of enzymatic mechanisms and operational parameters, may help bridge the gap between laboratory findings and large-scale implementation. Despite these challenges, bioremediation using white-rot fungi represents a promising and sustainable strategy for the treatment of textile effluents and the mitigation of environmental pollution.

## Figures and Tables

**Table 1:** Overview of white-rot fungal enzymatic systems and associated species.

Enzymes	Function	White-Rot Fungi Species	References
Aryl Alcohol Oxidase (AAO)	Supplies hydrogen peroxide for the peroxide enzymes’ activity through the oxidation of aromatic alcohols	*Bjerkandera adusta*, *Trametes versicolor*	[36,37]
Dye-decolorizing Peroxidase (DyP)	Oxidizes phenolic and non-phenolic aromatic compounds and participates in the decolorization of several dyes	*Bjerkandera adusta*, *Pleurotus ostreatus*	[38,39]
Laccase (Lac)	Oxidizes phenolic compounds and aromatic amines and reduces molecular oxygen to water	*Pleurotus eryngii*, *Pleurotus pulmonarius*, *Trametes hirsuta*, *Phlebia radiata*	[28,40,41,42]
Lignin Peroxidase (LiP)	Catalyzes the oxidative cleavage of non-phenolic lignin structures	*Phanerochaete chrysosporium*, *Phlebia radiata*	[43,44]
Manganese Peroxidase (MnP)	Oxidizes Mn^2+^ to Mn^3+^, which acts as a diffusible oxidant capable of degrading phenolic structures in lignin	*Bjerkandera adusta*, *Lentinus squarrosulus*, *Phanerochaete chrysosporium*, *Pleurotus eryngii*	[45,46,47,48]
Versatile Peroxidase (VP)	Displays combined catalytic properties of peroxidases and oxidases, oxidizing both phenolic and non-phenolic substrates	*Pleurotus eryngii*, *Pleurotus ostreatus*	[49,50]

**Table 2:** General trends in fungal enzymatic degradation of common dye classes.

Dye Class	Common Limitations	Operational Considerations	References
Azo	Formation of aromatic amines; incomplete mineralization	Often requires sequential anaerobic–aerobic processes or mediator systems	[81,82]
Anthraquinone	High structural stability; slower degradation rates	Higher-redox-potential enzymes are often required	[83,84]
Triarylmethane	Sensitivity to pH changes	Optimal activity is typically in acidic conditions	[85,86]
Reactive	High salinity in textile wastewater may inhibit enzymatic activity	Salinity and chloride levels should be considered	[87,88]
Several dye classes in real effluents	Presence of metals, salts, and auxiliary chemicals	Mediator addition and peroxide control may enhance efficiency	[17,89]

**Table 3:** Overview of mechanisms for biodegradation of dyes.

Biodegradation Mechanism	Description	Reactions Involved	References
Bioaccumulation	Dye bioaccumulation in fungi via intracellular accumulation, without chemical alteration. It depends on the dye’s affinity for functional groups on the cell membrane.	Transport across the cell membrane, absorption, in vacuoles or cell structures	[91,92]
Biosorption	Consists of the passive removal of dyes present in the medium through biosorption by fungal biomass.	Occurs primarily on the cell surface by adsorption through physicochemical interactions, such as ionic bonds, hydrogen bonds, and Van der Waals forces, without chemical alteration of the dye molecules	[91,92]
Enzymatic degradation	Direct enzymatic breakdown of dye molecules by extracellular and intracellular fungal enzymes, often resulting in biotransformation into less complex and toxic metabolites.	Oxidation, reduction and cleavage of chromophoric bonds (anthraquinone, azo)	[12,93]
Mineralization	Involves the complete degradation of the compound into simpler inorganic substances, typically following biotransformation by microorganisms.	Sequential oxidation reactions converting substrates into CO_2_, H_2_O and mineral salts	[94,95]

**Table 4:** Comparison of selected studies on fungal treatment of textile dyes using synthetic and real effluents.

Study	Treatment System	Effluent Type	Key Reported Outcomes
[108]	WRF *Trametes versicolor* producing extracellular oxidative enzymes (e.g., laccase) in batch cultures with glucose supplementation	Synthetic textile dye mixture and real textile wastewater from a dyeing facility	Up to 97% decolorization was achieved for synthetic dye mixtures, while ~92% removal was obtained for diluted real textile wastewater. More concentrated effluent showed lower efficiency (~40%), indicating inhibitory effects from wastewater constituents.
[109]	Immobilized WRF *Trametes hirsuta* PW17-41 producing MnP as the main ligninolytic enzyme	Textile dye wastewater containing mixed industrial dyes	The immobilized fungal system achieved ~95.4% decolorization within 48 h, associated with MnP activity of ~4942 U L^−1^. The immobilization matrix improved operational stability and allowed biomass to be reused for up to 12 treatment cycles while maintaining high dye removal efficiency.
[117]	Laccase enzyme isolated from *Pleurotus ostreatus*	Textile industrial effluent containing dyes (e.g., Turquoise VG, Black B, Yellow R, Methyl Red), heavy metals, and auxiliary chemicals	Enzymatic treatment promoted significant dye decolorization and reductions in key physicochemical parameters, including COD, biological oxygen demand (BOD), total dissolved solids (TDS), turbidity, and conductivity.
[102]	Biomass of the white-rot fungus *P. chrysosporium* used as a biosorbent for dye removal	Aqueous solutions containing Reactive Red and Reactive Blue dyes	Dead biomass removed ~82% of RR-198 and ~87% of RB-19 through biosorption. Optimal conditions included pH 3, 50 °C, 0.6 g adsorbent dosage, and 30 min contact time.

## Data Availability

No new data were created or analyzed in this study. Data sharing is not applicable to this article.
